# Aneurisma verdadeiro pós-traumático de artéria temporal

**DOI:** 10.1590/1677-5449.006615

**Published:** 2016

**Authors:** Ana Julia de Deus Silva, Ricardo Virginio dos Santos, Salvador José de Toledo Arruda Amato, Alexandre Campos Moraes Amato

**Affiliations:** 1 Universidade de Santo Amaro – UNISA, São Paulo, SP, Brasil.; 2 Amato – Instituto de Medicina Avançada, São Paulo, SP, Brasil.

**Keywords:** aneurisma, ferimentos, doenças vasculares

## Abstract

Os aneurismas de artéria temporal pós-traumático são eventos raros. Geralmente, são pseudoaneurismas. Como a causa mais frequente são ferimentos contusos, deve-se investigar todo paciente que possuir nodulação pulsátil na região da artéria temporal. O paciente apresentava protuberância pulsátil em região frontal direita há quatro meses, após queda de objeto pontiagudo, e o eco-Doppler evidenciou dilatação aneurismática. Assim, foi indicada sua excisão, que foi realizada com sucesso. O exame anatomopatológico demonstrou aneurisma verdadeiro traumático de artéria temporal superficial. Ocorrem devido ao fato de a artéria temporal superficial se localizar diretamente sobre o periósteo, o que a torna muito superficializada. Os aneurismas verdadeiros pós-traumáticos de artéria temporal são extremamente raros e podem ser confundidos com diversas outras afecções, como lipomas e cistos sebáceos.

## INTRODUÇÃO

O aneurisma pós-traumático de artéria temporal é considerado um evento raro, que normalmente é secundário a uma laceração ou uma ferida aberta e se apresenta mais frequentemente como falso aneurisma^1^. Ocorre em menos de 10% de todos os politraumatizados^2^. As causas mais frequentes são ferimentos contusos. Por isso, deve-se suspeitar dessa possibilidade em todos os pacientes que apresentam nódulo de consistência pulsátil no curso da artéria temporal após terem sofrido algum traumatismo^1^.

## RELATO DE CASO

**Figura 1:**
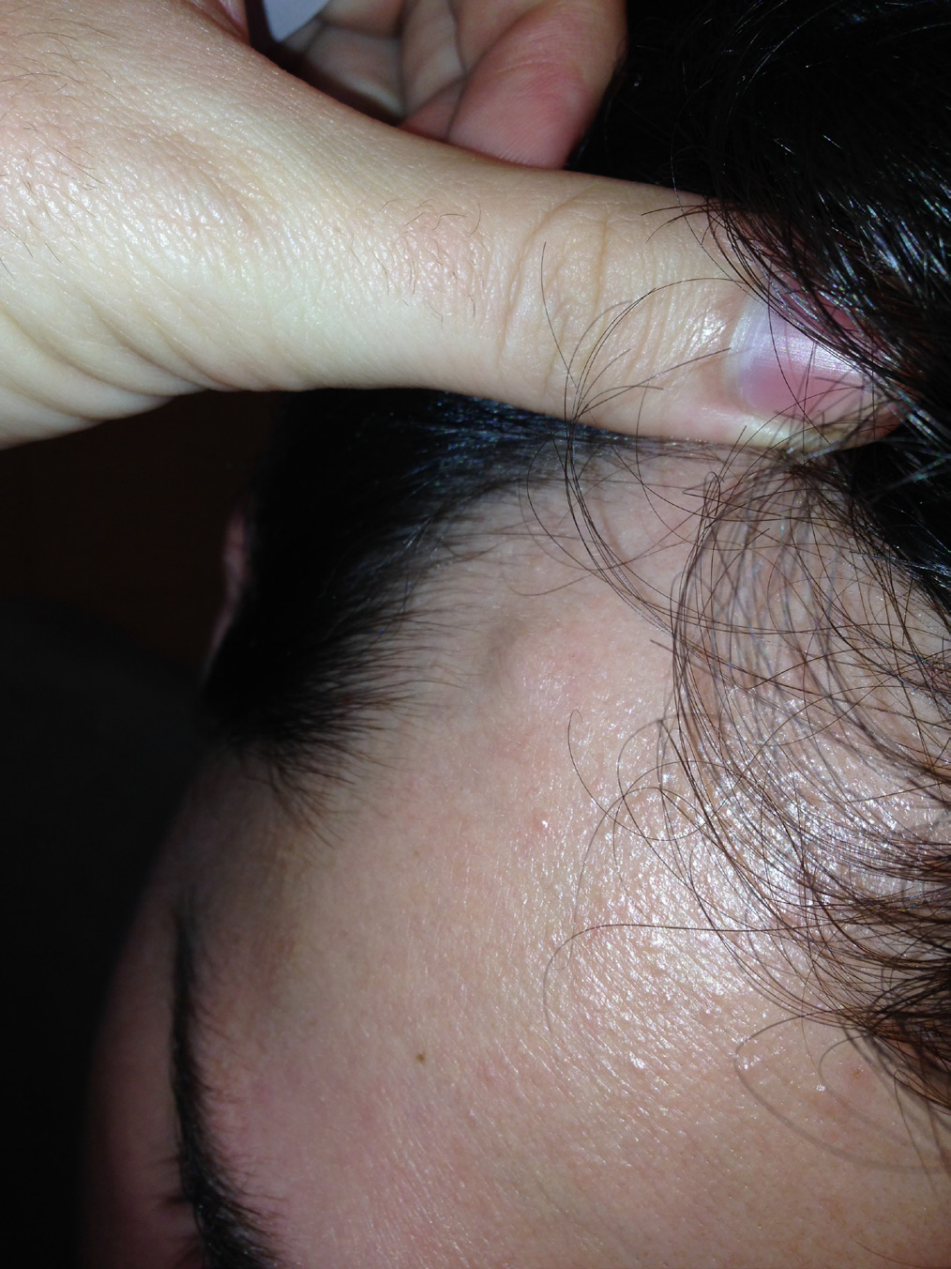
Protuberância em região temporal direita.

**Figura 2:**
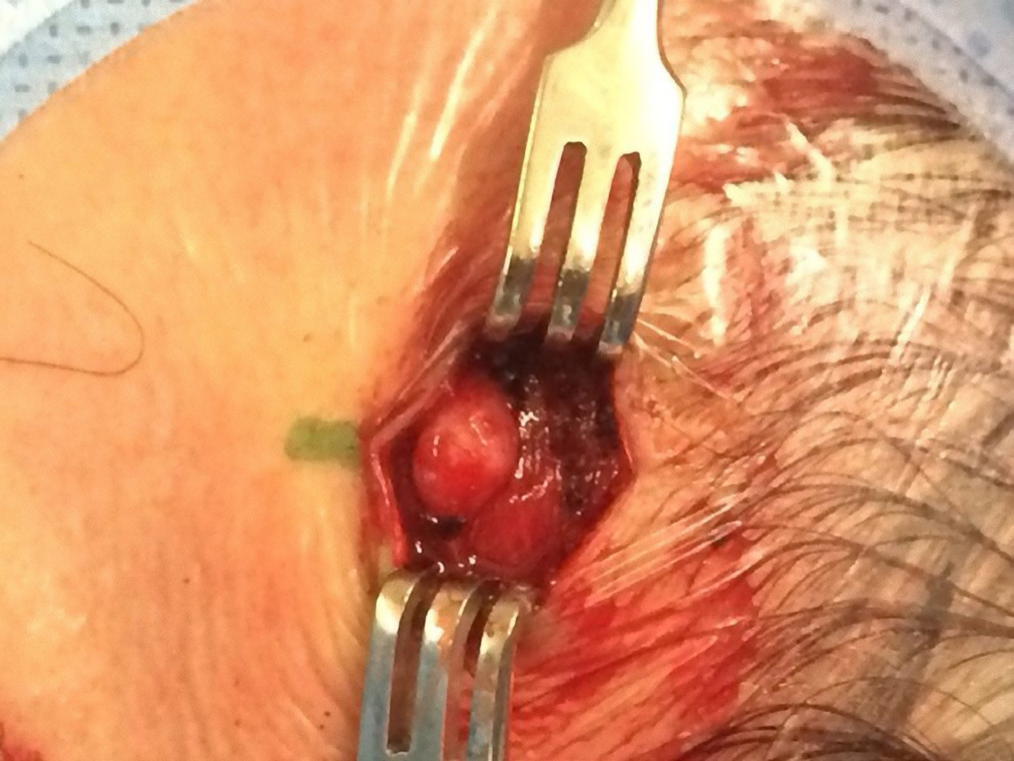
Ferida operatória que demonstra aneurisma de artéria temporal.

Paciente do sexo masculino, 25 anos, com queixa de protuberância em região frontal direita há quatro meses, após trauma por queda de objeto pontiagudo (Figura 1). Ao exame físico, o nódulo era pulsátil. Após a compressão da artéria temporal em região zigomática, o pulso desaparecia. O eco-Doppler evidenciava presença de segmento arterial de pequeno calibre em meio ao tecido celular subcutâneo, exibindo área de dilatação aneurismática com cerca de 7 x 6 x 3 mm e fluxo turbilhonado em seu interior, de padrão arterial. Foi realizada excisão da lesão com ligadura, o que evidenciou o aneurisma (Figura 2). O exame anatomopatológico demonstrou um aneurisma verdadeiro traumático de artéria temporal superficial com parede espessada, proliferação fibroblástica, focos de hemorragia prévia e neovascularização. O endotélio apresentava-se sem atipias, e havia ausência de granulomas e infiltrado inflamatório significativo.

## DISCUSSÃO

As lesões arteriais traumáticas possuem alta taxa de mortalidade e acarretam graves complicações. São causadas por traumatismo penetrante, fechado ou iatrogênico. As lesões podem levar a rotura arterial, hemorragia, oclusão arterial, dissecção e formação de pseudo e verdadeiro aneurisma ou de fístulas arteriovenosas^3^.

Os aneurismas de artéria temporal superficial são eventos raros, totalizando cerca de 200 casos relatados na literatura até o momento^4^. Os aneurismas de artéria temporal são, em 95% dos casos, de origem traumática e formam pseudoaneurismas. Os 5-8% restantes são aneurismas congênitos ou de origem aterosclerótica^5^^,^ 6. O aneurisma verdadeiro traumático, que envolve as três camadas do vaso acometido, é evento de extrema raridade. Acredita-se que os verdadeiros aneurismas podem se desenvolver através de uma afecção vascular preexistente, mas esta ainda é desconhecida^7^. O primeiro caso de aneurisma de artéria temporal foi descrito em 1740 por Thomas Bartolin^2^. Em 1934, Winslow e Edwards coletaram 108 casos de aneurisma de artéria temporal superficial, sendo 79 deles de origem traumática^2^.

Os aneurismas de artéria temporal pós-traumáticos ocorrem em função de seu ramo anterior se encontrar diretamente sobre o periósteo, o que o torna muito superficializado e mais propício a sofrer lesões com formação de tais eventos e fístulas arteriovenosas^8^. Acometem, então, o ramo frontal da artéria devido à sua exposição relativa^7^. Estão mais comumente associados a traumatismos cranianos e ocorrem após um trauma contuso de alta velocidade, surgindo de 2 a 6 semanas após a lesão^8^.

Devido à sua natureza pulsátil, o aneurisma de artéria temporal é facilmente identificado, mas deve haver um certo cuidado com os cistos presentes nessa região que não possuem pulso, pois podem ser aneurismas trombosados. Esses aneurismas são facilmente confundidos com lipomas, neuromas, nódulos, tumores císticos de glândula parótida^8^, fístulas arteriovenosas, hematomas, cistos sebáceos, abcessos e meningoceles, especialmente se a pulsatilidade do aneurisma é fraca^2^.

Os aneurismas de artéria temporal são indolores e possuem frêmito palpável^9^. O padrão-ouro para o seu diagnóstico é a tomografia computadorizada ou a angiorresonância^8^, mas um exame de grande aplicabilidade clínica é o eco-Doppler.

Caso o aneurisma não seja tratado, o paciente pode apresentar problemas estéticos, dores de cabeça e ruptura do mesmo^4^. A ressecção cirúrgica através de ligadura da artéria e exérese do aneurisma^2^ é o tratamento de escolha na maioria dos casos, mas outros tratamentos endovasculares, como injeção de trombina, que se mostra eficaz em 80% dos casos, e embolização por cateter, já foram descritos, sendo opções de tratamento quando não é possível realizar cirurgia ou por razões estéticas. O tratamento endovascular tem como desvantagem a possível formação de nódulo ou embolia da artéria carótida^4^.

## CONCLUSÃO

O aneurisma verdadeiro pós-traumático de artéria temporal é uma afecção extremamente rara^10^. Existem diversas outras doenças que podem confundir o cirurgião, como lipomas, cistos sebáceos, abcessos e outros^2^^,^ 7 ^,^. Diante do caso apresentado, pode-se concluir que a ressecção cirúrgica é o tratamento mais aconselhado, lembrando que sua exérese inadvertida pode ser catastrófica^2^^,^ 4.
