# Supplementary material for: Aneurisma verdadeiro pós-traumático de artéria temporal
Source: J Vasc Bras. 2016 Apr-Jun;15(2):165–7. [Article in Portuguese] doi: 10.1590/1677-5449.006615 (PMC5829713; doi:10.1590/1677-5449.006615)
Supplement: Translation

# Translation: True posttraumatic aneurysm of the temporal artery

**Authors:** Ana Julia de Deus Silva, Ricardo Virginio dos Santos, Salvador José de Toledo Arruda Amato, Alexandre Campos Moraes Amato

**Affiliations:** 1 Universidade de Santo Amaro – UNISA, São Paulo, SP, Brazil.; 2 Amato – Instituto de Medicina Avançada, São Paulo, SP, Brazil.

**Keywords:** aneurysm, wounds and injuries, vascular diseases

## Abstract

Posttraumatic aneurysms of the temporal artery are rare events and are generally pseudoaneurysms. Since the most frequent cause is blunt injury, all patients with a pulsating nodule in the region of the temporal artery should be investigated. This patient presented with a pulsating protuberance in the right frontal area with onset 4 months previously after being hit by a falling sharp object. Doppler ultrasonography showed evidence of aneurysmal dilatation, which was excised successfully. Pathology results demonstrated a true traumatic aneurysm of the superficial temporal artery. They occur because the superficial temporal artery is located directly over the periosteum, meaning it is very superficial. True posttraumatic aneurysms of the temporal artery are extremely rare and may be confused with many other conditions, such as lipomas and sebaceous cysts.

## INTRODUCTION

Posttraumatic aneurysm of the temporal artery is considered a rare event, which normally follows a laceration or open wound and the most common presentation is a false aneurysm.^1^ The condition occurs in less than 10% of all multiple injury cases.^2^ The most common causes are blunt injuries. This possibility should therefore be suspected in all patients who present with a pulsating nodule in the region of the temporal artery after a traumatism.^1^

## CASE REPORT

A 25-year-old male patient presented with a pulsating protuberance in the right frontal area with onset 4 months previously after being hit by a falling sharp object (Figure 1). On physical examination the nodule was found to be pulsating. The pulsation ceased on compression of the temporal artery in the zygomatic region. Doppler ultrasonography revealed a small-caliber arterial segment within the subcutaneous cell tissue, exhibiting an aneurysmal dilatation area of approximately 7 x 6 x 3 mm and turbulent flow inside, with an arterial flow pattern. The lesion was excised and ligated and found to be an aneurysm (Figure 2). Pathology results showed that it was a true traumatic aneurysm of the superficial temporal artery with a thickened wall, fibroblastic proliferation, foci of prior hemorrhage and neovascularization. The endothelium was free from atypical phenomena and there were no granulomas or significant inflammatory infiltrate.

**Figure 1:** Protuberance in the right temporal region.

**Figure 2:** Surgical wound revealing an aneurysm of the temporal artery.

## DISCUSSION

Traumatic arterial injuries have a high mortality rate and lead to severe complications. They can be caused by penetrating or blunt traumas or may be iatrogenic. Injuries can lead to rupture of arteries, hemorrhage, arterial occlusion, dissection and formation of pseudoaneurysms, true aneurysms or arteriovenous fistulas.^3^

Aneurysms of the superficial temporal artery are rare events and there are currently a total of around 200 cases reported in the literature.^4^ In 95% of cases, aneurysms of the temporal artery have traumatic origin and form pseudoaneurysms. The remaining 5-8% are congenital aneurysms or have atherosclerotic origin.^5^^,^ 6 A true traumatic aneurysm, involving all three layers of the vessel, is an extremely rare event. It is believed that true aneurysms may develop from a preexisting vascular condition, but this remains uncertain.^7^ The first case of temporal artery aneurysm was described in 1740 by Thomas Bartolin.^2^ In 1934, Winslow and Edwards collected 108 cases of superficial temporal artery aneurysm, 79 of which had a traumatic origin.^2^

Posttraumatic aneurysms of the temporal artery occur because the anterior branch of the artery is located directly over the periosteum, which means it is very superficial and prone to injury with formation of aneurysms and arteriovenous fistulas.^8^ They therefore involve the frontal branch of the artery, because it is relatively more exposed.^7^ They are more commonly related to head traumas and they occur after a high velocity blunt trauma, appearing from 2 to 6 weeks after the injury.^8^

An aneurysm of the temporal artery is easily identified by its pulsating nature, but a certain degree of care must be taken with cysts with no pulse in this region, since they may actually be thrombosed aneurysms. These aneurysms are easily confused with lipomas, neuromas, nodules, cystic tumors of the parotid gland,^8^ arteriovenous fistulas, hematomas, sebaceous cysts, abscesses and meningocele, especially if the aneurysm’s pulse is weak.^2^

Aneurysms of the temporal artery are painless and have a palpable thrill.^9^ The gold standard for diagnosis is computed tomography or magnetic resonance angiography,^8^ but Doppler ultrasonography has great clinical applicability.

If the aneurysm is not treated, the patient can suffer esthetic problems, headaches and aneurysmal rupture.^4^ Surgical resection with ligature of the artery and excision of the aneurysm^2^ is the treatment of choice in the majority of cases, but other endovascular treatments, such as injection of thrombin, which proves effective in 80% of cases, and embolization by catheter have been described, and are treatment options when it is not possible to treat with surgery or for esthetic reasons. The disadvantage of endovascular treatment is the possibility of formation of a nodule or embolism of the carotid artery.^4^

## CONCLUSIONS

True posttraumatic aneurysm of the temporal artery is an extremely rare condition.^10^ There are many other diseases that can confuse the surgeon, such as lipomas, sebaceous cysts, abscesses and others.^2^^,^ 7 From the case described here it can be concluded that surgical resection is the most advisable treatment, bearing in mind that inadvertent exceresis can be catastrophic.^2^^,^ 4
